# “Quinone Millipedes” Reconsidered: Evidence for a Mosaic-Like Taxonomic Distribution of Phenol-Based Secretions across the Julidae

**DOI:** 10.1007/s10886-016-0680-4

**Published:** 2016-03-14

**Authors:** Michaela Bodner, Boyan Vagalinski, Slobodan E. Makarov, Dragan Ž. Antić, Ljubodrag V. Vujisić, Hans-Jörg Leis, Günther Raspotnig

**Affiliations:** Institute of Zoology, University of Graz, Universitätsplatz 2, 8010 Graz, Austria; Institute of Biodiversity and Ecosystem Research, Department of Animal Diversity and Resources, Bulgarian Academy of Sciences, 2 Gagarin Street, 1113 Sofia, Bulgaria; Institute of Zoology, Faculty of Biology, University of Belgrade, Studentski Trg 16, Belgrade, 11000 Serbia; Faculty of Chemistry, University of Belgrade, Studentski Trg 12-16, Belgrade, 11000 Serbia; Research Unit of Osteology and Analytical Mass Spectrometry, Medical University, University Children’s Hospital, Auenbruggerplatz 30, 8036 Graz, Austria

**Keywords:** Chemical defense, Quinone millipedes, Phenols, Julida, Julidae, Cylindroiulini, Brachyiulini, Leptoiulini, Diplopoda

## Abstract

The defensive chemistry of juliformian millipedes is characterized mainly by benzoquinones (”quinone millipedes”), whereas the secretions of the putative close outgroup Callipodida are considered to be exclusively phenolic. We conducted a chemical screening of julid secretions for phenolic content. Most species from tribes Cylindroiulini (15 species examined), Brachyiulini (5 species examined), Leptoiulini (15 species examined), Uncigerini (2 species examined), Pachyiulini (3 species examined), and Ommatoiulini (2 species examined) had non-phenolic, in most cases exclusively benzoquinonic secretions. In contrast, tribes Cylindroiulini, Brachyiulini, and Leptoiulini also contained representatives with predominantly phenol-based exudates. In detail, *p*-cresol was a major compound in the secretions of the cylindroiulines *Styrioiulus pelidnus* and *S. styricus* (*p*-cresol content 93 %) and an undetermined *Cylindroiulus* species (*p*-cresol content 51 %), in the brachyiulines *Brachyiulus lusitanus* (*p*-cresol content 21 %) and *Megaphyllum fagorum* (*p*-cresol content 92 %), as well as in an undescribed *Typhloiulus* species (*p*-cresol content 32 %, Leptoiulini). In all species, *p*-cresol was accompanied by small amounts of phenol. The secretion of *M. fagorum* was exclusively phenolic, whereas phenols were accompanied by benzoquinones in all other species. This is the first incidence of clearly phenol-dominated secretions in the Julidae. We hypothesize a shared biosynthetic route to phenols and benzoquinones, with benzoquinones being produced from phenolic precursors. The patchy taxonomic distribution of phenols documented herein supports multiple independent regression events in a common pathway of benzoquinone synthesis rather than multiple independent incidences of phenol biosynthesis.

**Electronic supplementary material:**

The online version of this article (doi:10.1007/s10886-016-0680-4) contains supplementary material, which is available to authorized users.

## Introduction

Millipedes are well protected against predators. Apart from hardened cuticles, disturbance-related coiling behaviors, or defensive bristles (Polyxenida), the majority of millipedes are known to release repellent and noxious fluids from serial exocrine glands, serving as an effective means of active defense. Depending on the taxonomic group, diplopod defensive secretions show considerable chemical diversity as comprehensively reviewed recently (Shear 2015). Briefly, Glomerida and Polyzoniida produce unique alkaloids (e.g., Meinwald et al. 1966, 1975; Schildknecht et al. 1966; Shear et al. 2011; Wood et al. 2000), Polydesmida produce cyanogenic compounds (Makarov et al. 2011; Shear et al. 2007), whereas all groups of Juliformia mainly secrete quinones (e.g., Eisner et al. 1978; Vujisić et al. 2011). Indeed, the latter group - the Juliformia - have been known as “quinone millipedes” (Eisner et al. 1978), implying that this superorder almost exclusively produces quinone-based secretions. Interestingly, within the putative juliformian sister group “Polydesmida (Chordeumatida (Stemmiulida + Callipodida))” several taxa rely on phenolic secretions (e.g., Blanke and Wesener 2014; Shear 2015). Callipodids, for instance, appear to exclusively produce phenols (e.g., Makarov et al. 2011), which give them a characteristic and obtrusive odor, easily noticeable by humans over distances of several meters. Additionally, stemmiulidans (Shear 2015) and several polydesmidans have been reported to discharge phenolics, and in both cases the most frequently occurring component was *p*-cresol (Duffey et al. 1977; Mori et al. 1994; Noguchi et al. 1997; Shear et al. 2007; Taira et al. 2003).

In many Arthropoda, the biosynthesis of phenols and benzoquinones is thought to be related. There are good examples of arthropods that utlize both phenolic and benzoquinonic compounds (e.g., Rocha et al. 2013). In laniatorean harvestmen, for instance, the secretions of representatives of basal grassatorean families rely on phenolics, whereas the secretions of derived grassatoreans may contain both phenols and benzoquinones or may even be purely benzoquinonic (Föttinger et al. 2010; Raspotnig et al. 2015). In laniatoreans, benzoquinones appear to present a derived character state, that can be produced from phenols by para-oxidation, and the production of benzoquinones may be considered an extension of the pathway to phenols (Raspotnig et al. 2015; Rocha et al. 2013).

In the scope of our studies on the evolutionary history of secretion chemistry in diplopods, it is essential to pinpoint basic correlations between the chemistry of different taxa. We here hypothesize that benzoquinones in juliformians evolved from the ancestral state of phenolic secretions, as still present in juliformian outgroups (Raspotnig and Bodner 2014). To test our idea, we conducted extensive chemical screening of julid secretions for their phenolic content. So far, phenolics in juliformians have been regarded to be an exception or even completely missing: only one representative of Julida, namely a member of the basal julidan family Parajulidae, *Oriulus venustus* (Wood 1864), was reported to discharge a phenol-rich secretion (Kluge and Eisner 1971). There are, however, an increasing number of reports on julid phenolics as minor, trace, or by-products of the quinone-rich secretions of certain species (e.g., Sekulić et al. 2014).

We provide evidence that phenol-based secretions are more widespread in the Julidae, and that phenolics even prevail over quinones in some species of the tribes Cylindroiulini, Brachyiulini, and Leptoiulini.

## Methods and Materials

### Collection of Species

Individuals, mostly adults, from 42 species belonging to 17 genera and 6 tribes of Julidae and 2 species of Blaniulidae (see supplemental Table S1) were collected by hand, either from the leaf litter layer or from deeper soil and breakstone beneath the leaf litter layer at various locations in Austria, Germany, Italy, France, Spain, Bulgaria, Serbia, and Azerbaijan. Vouchers were deposited at the Natural History Museum of Vienna (NHMW), Natural History Museum of Denmark (ZMUC), Hungarian Natural History Museum (HNHM), National Museum of Natural History Sofia (NMNHS), and at the Faculty of Biology, Institute of Zoology, University of Belgrade (FBIZO).

### Extraction and Analysis of Defensive Secretions

Secretions were obtained by whole body extraction of single individuals in hexane for 15 min. The defensive secretions were discharged directly into the solvent. Aliquots of diluted extracts (1.5 μl) were analyzed by gas chromatography– mass spectrometry (GC-MS), using a trace gas chromatograph coupled to a DSQ I mass spectrometer (MS), both from Thermo (Vienna, Austria). GC and MS conditions were the same as previously described (Bodner and Raspotnig 2012). Gas chromatographic retention indices (RI) of extract components were calculated using an alkane standard mixture (Van den Dool and Kratz 1963).

High-resolution mass spectrometry was carried out on a Q-exactive high-resolution Orbitrap MS from Thermo (Vienna, Austria). Samples containing secretion in hexane were gently reduced under nitrogen, then dissolved in methanol: water (1:1) with 1 % formic acid, and analyzed by direct infusion ESI-MS and HPLC-MS, respectively. Components were observed as [M + H]^+.^- ions as well as Na - and K - adducts.

### Reference Compounds and Derivatization

For comparison of GC-MS data to authentic reference compounds 1,4-benzoquinone, 2,3-dimethoxy-5-methyl-1,4-benzoquinone, *p*-cresol, *o*-cresol, and *m*-cresol, methyl-paraben, and 2-phenylphenol were purchased from Sigma (Vienna, Austria). 2,3,5,6-Tetramethoxy-1,4-benzoquinone was from MicroCombiChem (Wiesbaden, Germany), and 2,3-dimethoxy-5-methyl-1,4-hydroquinone from abcr GmbH & Co KG (Karlsruhe, Germany). As reference for other compounds, particularly 2-methyl-1,4-benzoquinone, 2-hydroxy-3-methyl-1,4-benzoquinone, 2-methoxy-3-methyl-1,4-benzoquinone, 2,3-dimethoxy-1,4-benzoquinone, 2-methylhydroquinone, we used natural sources from which these compounds had already been identified (*Allajulus dicentrus*: Bodner and Raspotnig 2012; *Cylindroiulus boleti*: Vujisić et al. 2011).

Derivatization of cresol isomers to their corresponding trimethylsilyl (TMS)-ethers was conducted by adding 75 μl N-methyl-N-(trimethylsilyl)-trifluoracetamid (MSTFA in pyridine 2:1; with 1 % trimethylchlorosilane [TMCS]) to 50 μl of secretion in hexane. After 30 min of reaction at 55 °C, an aliquot of the mixture (1.5 μl) was injected directly into the GC-MS.

### Profile Evaluation and Statistics

Relative abundance of single components (in %) was calculated by integration of peak areas in the chromatograms, leading to individual secretion profiles. This semi-quantitative method to calculate secretion profiles is widely used in chemosystematic studies of different arthropod taxa (e.g., mites (Heethoff 2012; Sakata et al. 2003); opilionids (Hara et al. 2005); thrips (Suzuki et al. 1989); diplopods (Makarov et al. 2010)). Individual chromatographic profiles were compared eventually by non-metric multidimensional scaling (NMDS) using the Bray-Curtis coefficient of dissimilarity (Bray and Curtis 1957).

## Results

### Phenol-Producing Julid Species

From the 42 species of brachyiuline, cylindroiuline, leptoiuline, uncigerine, pachyiuline, and ommatoiuline Julidae, the secretions of 28 species were investigated for the first time; the same is true for the 2 blaniulids (see Table 1). For the remaining 14 species, literature data from previous investigations were available – these species, however, were re-investigated. Phenol-rich secretions, i.e., phenolic content >20 % of the whole secretion (based on comparison of peak areas), were found for 6 species from 3 different julid tribes: (i) *Brachyiulus lusitanus* and *Megaphyllum fagorum* (both Brachyiulini), (ii) *Styrioiulus styricus*, *S. pelidnus*, and *Cylindroiulus* sp. (Cylindroiulini), and (iii) a yet undescribed species of genus *Typhloiulus* (Leptoiulini).

**Table 1:** Chemical classes in the defensive secretions of Julida

family		species		phenols	quinones	other
Blaniulidae		***Blaniulus***	***dollfusi***	−	+	+
			*guttulatus* *	−	+	+
		*Cibiniulus*	*phlepsii* *	−	+	+
		*Nopoiulus*	*kochii* *	−	+	+
		***Proteroiulus***	***fuscus***	−	+	+
Julidae	Brachyiulini	*Anaulaciulus*	*okinawaensis* *	−	+	−
			*sp.* *	−	+	+
		***Brachyiulus***	***lusitanus***	+	+	+
		***Megaphyllum***	***bosniense*** *	−	+	−
			***fagorum***	+	−	−
			***hercules***	−	+	+
			***silvaticus***	−	+	+
			*unilineatum* *	−	+	−
	Cylindroiulini	***Allaiulus***	***dicentrus*** *	−	+	+
			***molybdinus***	−	+	+
			*nitidus* *	−	+	−
		***Cylindroiulus***	***apenninorum***	−	+	+
			***boleti*** *	−	+	−
			***broti***	−	+	+
			***caeruleocinctus*** *	−	+	+
			*londinensis* *	−	+	−
			***luridus*** *	−	+	−
			***meinerti*** *	−	+	+
			**sp. (present study)**	+	+	−
		***Enantiulus***	***karawankianus***	−	+	+
			***nanus*** *	−	+	+
			***transsilvanicus***	−	+	+
		***Kryphioiulus***	***occultus***	−	+	+
		***Styrioiulus***	***pelidnus***	+	+	−
			***styricus***	+	+	−
	Iulini	*Julus*	*scandinavius* *	−	+	+
	Leptoiulini	***Lamellotyphlus***	***sotirovi***	−	+	?
		***Leptoiulus***	***proximus*** *	−	+	+
			***trilineatus*** *	−	+	−
		***Ophiulus***	***pilosus*** *	−	+	−
		***Typhloiulus***	***spec.nov.***	+	+	+
			***bureschi***	−	+	+
			***georgievi***	−	+	+
			***lobifer***	−	+	?
			***nevoi***	−	+	?
			***orpheus***	−	+	+
			***serborum***	+	+	?
		***Serboiulus***	***deelemani***	+	+	?
			***kresnik***	−	+	?
			***lucifugus***	−	+	?
		***Xestoiulus***	***imbecillus***	−	+	+
	Uncigerini	***Unciger***	***foetidus*** *	−	+	−
			***transsilvanicus*** *	+	+	+
	Pachyiulini	***Dolichoiulus***	***hercules***	−	+	+
		***Pachyiulus***	***cattarensis***	−	+	+
			***hungaricus*** *	−	+	+
	Ommatoiulini	***Ommatoiulus***	***bipartitus***	−	+	+
			***sabulosus*** *	−	+	+
		*Tachypodoiulus*	*niger* *	−	+	+
Parajulidae		*Oriulus*	*delus* *	+	+	−
		*Uroblaniulus*	*canadensis* *	−	+	−

Only species in bol were investigated in this study. For species marked with * literature data are available (see Shear 2015; Vujisić et al. 2011, 2014). Species in bold and marked with * were reinvestigated

### Compound Identification

The major phenolic compound in all 6 species appeared to be a methylated phenol isomer (= cresol: peak D; M^+^ at *m*/*z* 108). The mass spectra of the three possible cresol isomers (*p*-, *o*-, *m*-cresol) are basically indistinguishable, at best showing slight differences in the intensity of M^+^ (at *m*/*z* 108) and M-1^+^-ions (at *m*/*z* 107), respectively. The retention index measured for compound D (RI = 1071) was clearly different from authentic *o*-cresol (RI = 1051), but corresponded to both the RI of authentic *m*-cresol (RI = 1072) as well *p*-cresol (RI = 1071). TMS-Derivatization of compound D, *p*- and *m*-cresol, respectively, led to cresol-TMS ethers, which again showed indistinguishable mass spectra: M^+^ at *m*/*z* 180 (45), fragment ions at *m*/*z* 165 (100), 149 (5), 135 (6), 105 (3), 91 (9). The retention time of the TMS-derivative of compound D, however, corresponded to *p*-cresol-TMS ether only (*m*-cresol-TMS: RI = 1151; *p*-cresol-TMS: RI = 1160; compound D-TMS: RI = 1160). The second phenolic compound of the extracts (peak B: M^+^ at *m*/*z* 94) was identified as phenol. Quantitatively, *p*-cresol accounted for about 20 % (*B. lusitanus*) up to more than 90 % of the secretions (*Styrioiulus*, *M. fagorum*), whereas phenol generally was a minor or even a trace compound in all 6 species. Only the secretion of *M. fagorum* was purely phenolic.

**Fig. 1:**
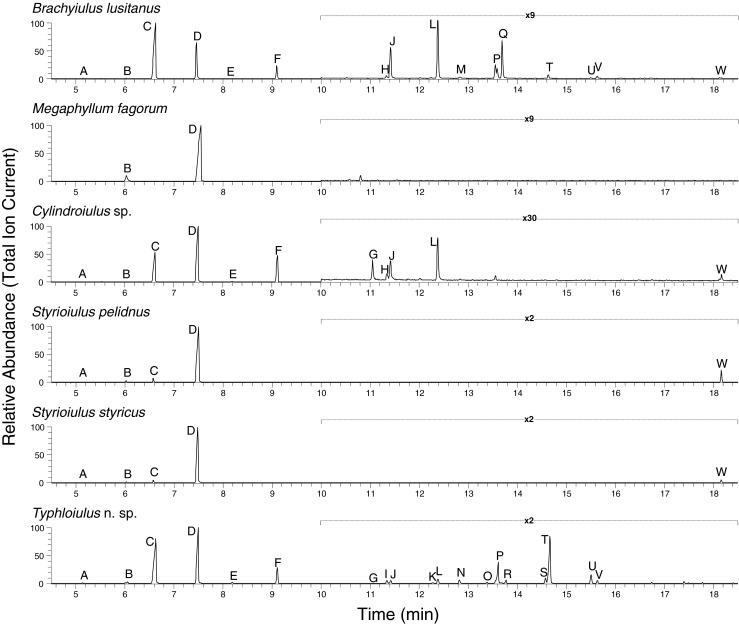
Gas chromatographic profiles of the defensive secretions of phenol-producing julid species. Phenolic compounds are: peak B (phenol), peak D (*p*-cresol). For a complete list of compounds see Table 2. Note that the chromatographic response between 10.00 and 18.50 min retention time is amplified for a better demonstration of minor peaks

From the secretions of *B. lusitanus*, *Cylindroiulus* sp., *S. pelidnus*, *S. styricus*, and *Typhloiulus* n. sp. additional 21 components, mainly benzoquinones, could be separated (Table 2, Fig. 1). Fourteen of the compounds were fully or at least partly identified, either by a comparison of GC-MS data to authentic standards, by a comparison of mass spectra and retention indices to data from literature, or by high resolution mass spectrometry, respectively (Table 2). These compounds comprised 1,4-benzoquinone and differentially substituted methyl-, hydroxy-, and methoxy-1,4-benzoquinones (peaks A, C, E, F, G, I, J, L, and M) as well as two hydroquinones (peaks H, M), all of which had been described previously from the secretions of various Juliformia (Shear 2015).

**Table 2:** Gas chromatographic and mass spectral data

Peak no.	Retention index RI measured/authentic reference		Mass spectrometric fragmentation *m*/*z* (rel. Intensity)	Identified as
A	920	920	110 ([M + 2]^+^, 32), 108 (M^+^,100), 82 (42), 80 (39), 54 (82)	1,4-benzoquinone
B	977	978^(a)^	95 ([M + 1]^+^, 7), 94 (M^+^,100), 66 (35), 65 (24), 55 (6)	phenol
C	1014	1014	122 (M^+^, 100), 94 (99), 82 (64), 68 (35), 66 (53), 54 (67)	2-methyl-1,4-benzoquinone
D	1071	1071	108 (M^+^, 82), 107 (100), 90 (10), 80 (14), 79 (24), 77 (29)	p-cresol
E	1119	1120	138 (M^+^,100), 137 (3), 110 (7), 83 (6), 82 (14), 54 (10)	2-hydroxy-3-methyl-1,4-benzoquinone
F	1182	1183	152 (M^+^, 100), 151 (21), 137 (5), 123 (7), 122 (43), 109 (14), 94 (5), 83 (8), 82 (10), 81 (7), 67 (7), 66 (15), 54 (10), 53 (12)	2-methoxy-3-methyl-1,4-benzoquinone
G	1319	1320	170 ([M + 2]^+^, 16), 168 (M^+^, 100), 155 (7), 153 (39), 140 (1), 138 (6), 125 (13), 123 (88), 122 (13), 112 (2), 95 (11), 94 (5), 82 (10), 69 (25), 54 (12)	2,3-dimethoxy-1,4-benzoquinone
H	1341	1341	124 (M^+^,100), 123 (42), 107 (8), 105 (4), 95 (15), 77 (6), 69 (7), 67 (10), 57 (10)	2-methylhydroquinone
I	1341	-	152 (M^+^,100), 137 (11), 124 (36), 123 (26), 122 (22), 109 (8), 94 (7), 84 (11), 69 (83), 66 (25), 56 (9)	2-methoxy-5-methyl-1,4-benzoquinone^(b)^
J	1346	-	152 (M^+^, 68), 137 (14), 124 (75), 123 (19), 122 (66), 109 (22), 96 (15), 94 (12), 81 (6), 69 (100), 66 (27), 53 (26)	2-methoxy-6-methyl-1,4-benzoquinone^(b)^
K	1411	-	154 (M^+^, 100), 139 (6), 128 (6), 126 (58), 111 (20), 98 (12), 97 (16), 85 (8), 82 (39), 72 (9), 57 (22), 54 (27)	2-hydroxy-3-methoxy-1,4-benzoquinone^(c)^
L	1419	1420	182 (M^+^, 97), 167 (42), 153 (16), 139 (24), 137 (100), 136 (21), 121 (7), 111 (19), 108 (7), 96 (7), 83 (24), 69 (13), 68 (17), 67 (7)	2,3-dimethoxy-5-methyl-1,4-benzoquinone
M	1455	-	184 (M^+^, 95), 169 (83), 152 (45), 126 (24), 121 (100), 93 (23), 69 (13), 65 (15)	mixed spectrum: 2,3-dimethoxy-5-methylhydroquinone + methyl-paraben^(d)^
N	1455	1456	152 (M^+^, 50), 122 (6), 121 (100), 93 (16), 92 (3), 65 (7)	methyl-paraben (*p*-hydroxybenzoic acid methyl ester)
O	1499	-	184 (M^+^, 52), 169 (100), 140 (7), 139 (12), 127 (14), 123 (25), 113 (23), 87 (18), 85 (42), 72 (11), 69 (7), 68 (6), 54 (6)	dimethoxy-hydroxy-benzoquinone isomer^(c)^
P	1518	-	168 (M^+^, 100), 153 (21), 140 (61), 125 (22), 112 (7), 97 (16), 96 (10), 85 (20), 72 (12), 68 (33)	2-hydroxy-3-methoxy-5-methyl-1,4-benzoquinone^(c)^
Q	1524	1526	170 (M^+^, 100), 169 (78), 142 (12), 141 (36), 139 (10), 115 (21), 89 (3)	2-phenylphenol
R	1532	-	214 (M^+^, 100), 169 (11), 168 (35), 167 (12), 155 (19), 138 (51), 137 (21), 136 (17), 127 (75), 114 (6), 99 (26), 68 (17), 59 (17)	unidentified
S	1598	-	200 (M^+^, 100), 185 (10), 151 (42), 139 (7), 123 (12), 85 (2), 69 (3)	unidentified
T	1606	-	198 (M^+^, 100), 183 (86), 170 (2), 165 (28), 155 (15), 153 (9), 152 (4), 137 (32), 127 (20), 123 (4), 109 (8), 99 (26), 96 (6), 87 (19), 83 (5), 72 (4), 68 (9)	dimethoxy-hydroxy-methyl-benzoquinone isomer 1^(c)^
U	1680	-	198 (M^+^, 100), 183 (18), 180 (4), 170 (8), 165 (6), 155 (26), 152 (7), 140 (3), 137 (4), 127 (12), 125 (3), 112 (5), 109 (8), 99 (18), 85 (12), 83 (12), 72 (4)	dimethoxy-hydroxy-methyl-benzoquinone isomer 2^(c)^
V	1691	-	198 (M^+^, 100), 183 (55), 180 (11), 170 (20), 169 (18), 165 (7), 155 (11), 151 (5), 140 (2), 137 (4), 127 (14), 125 (37), 123 (1), 112 (7), 109 (2), 99 (33), 85 (26), 83 (24), 82 (1), 72 (1), 67 (4)	dimethoxy-hydroxy-methyl-benzoquinone isomer 3^(c)^
W	1932	-	230 ([M + 2]^+^, 59), 228 (M^+^,100), 213 (19), 211 (11), 199 (15), 185 (24), 159 (13), 129 (19), 119 (27), 110 (17), 91 (35), 69 (30), 65 (31), 53 (26)	C_14_H_13_O_3_^(e)^

^a^Radulović et al. (2010). ^b^ Isomers assigned according to Wu et al. (2007). ^c^ Tentatively identified on the basis of mass spectral fragmentation and high resolution mass spectrometry. ^d^ Peak M was specific for *Brachyiulus lusitanus*, containing 2 compounds, namely 2,3-dimethoxy-5-methylhydroquinone and methyl-paraben in a ratio of about 1:1. RIs measured for authentic 2,3-dimethoxy-5-methylhydroquinone and methyl-paraben, respectively, proved to be indistinguishable (for both compounds RI 1455), and co-injection of both compounds (in 1:1 ratio) led to a single, sharp peak, showing a mass spectrum as observed for peak M. On the other hand, methyl-paraben (without 2,3-dimethoxy-5-methylhydroquinone) was found in the extracts of *Typhloiulus* n. sp. (peak N). ^e^ Molecular formula, based on high resolution mass measurement

All other compounds (K, N, O, P, Q, R, S, T, U, V, and W) were, to our knowledge, new for millipede secretions. Compounds N and Q were identified as methyl-paraben and 2-phenylphenol, respectively. Compound W was partly identified: it exhibited a molecular ion at *m*/*z* 228 (base peak), together with an intense M + 2-ion, indicating the reduction of a quinone in the hot injector. The compound was first suspected to represent a 2,3,5,6-tetramethoxy-1,4-benzoquinone (TM-BQ, C_10_H_12_O_6_), but a comparison to authentic TM-BQ showed distinctly different retention times and RIs, respectively (measured RI_TM-BQ_ = 1713; measured RI_compound W_ = 1932). High resolution mass spectrometry via HPLC-MS led to a probable elemental composition of C_14_H_12_O_3_ (measured monoisotopic mass: 228.0786; *R* = 140.000; theoretical monoisotopic mass: 228.0786). This particular molecular formula indicates a highly condensed component (rings plus double bonds =9), and thus, most likely 2 ring systems. The detailed structure of this compound remained unknown. Compounds K, O, P, T, U, and V were not fully identified. These compounds shared the mass spectrometric characteristics of hydroxy-methoxy-1,4-benzoquinones (Budzikiewicz et al. 1967: p. 530). Possible structures, supported by high resolution mass spectrometry, are proposed in Table 2. The detailed chemical identification of these compounds will be presented in a separate paper. Compounds R and S remained unidentified.

### Secretion Profiles

**Fig. 2:**
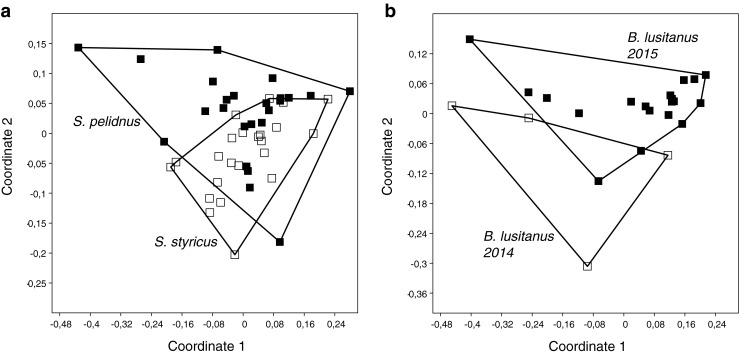
Statistical discrimination of chemical profiles of (**a**) individuals of *Styrioiulus pelidnus* and *S. styricus*, and (**b**) individuals of *Brachyiulus lusitanus* from 2 collections. Plots based on NMDS using the Bray Curtis coefficient

The 6 phenol-producing species exhibited highly consistent secretion profiles in terms of profile quality (= compound composition) as well as relative abundance of components. The chromatographic profiles are given in Table 3. Only the two species of *Styrioiulus* showed indistinguishable profiles, and a comparison of individual profiles based on non-metric multidimensional scaling (NMDS) resulted in a scatter plot, showing one single cluster (Fig. 2a). All other species were readily distinguishable by their characteristic secretion chemistry. For *B. lusitanus*, individuals from the same location exhibited slightly different but statistically distinguishable secretion patterns (Fig. 2b) in a repeated investigation after a time interval of one year. Statistically significant differences between female and male secretion pattern were not observed for any of the species.

**Table 3:** Gas chromatographic profiles* of defensive secretions of phenol-producing Julidae

Peak no.	compound	*Brachyiulus lusitanus*	*Megaphyllum fagorum*	*Cylindroiulus sp.*	*Styrioiulus pelidnus*	*Styrioiulus styricus*	*Typhloiulus n.sp.*
A	1,4-benzoquinone	trace	-	0.2	0.1 ± 0.1	0.1 ± 0.1	0.1 ± 0.1
B	**phenol**	**trace**	**7.8**	**0.1**	**1.3 ± 0.5**	**2.1 ± 0.8**	**0.7 ± 0.1**
C	2-methyl-1,4-benzoquinone	54.3 ± 6.2	-	25.2	3.5 ± 1.1	3.8 ± 1.0	31.8 ± 4.2
D	***p*** **-cresol**	**21.4 ± 9.7**	**92.2**	**50.6**	**93.1 ± 2.0**	**93.0 ± 1.6**	**31.8 ± 6.3**
E	2-hydroxy-3-methyl-1,4-benzoquinone	0.5 ± 0.5	-	0.5	-	-	0.4 ± 0.1
F	2-methoxy-3-methyl-1,4-benzoquinone	16.2 ± 10.7	-	20.9	-	-	8.1 ± 1.8
G	2,3-dimethoxy-1,4-benzoquinone	-	-	0.5	-	-	trace
H	2-methylhydroquinone	0.1 ± 0.1	-	0.2	-	-	-
I	2-methoxy-5-methyl-1,4-benzoquinone	-	-	-	-	-	0.7 ± 0.2
J	2-methoxy-6-methyl-1,4-benzoquinone	1.9 ± 0.8	-	0.5	-	-	0.6 ± 0.2
K	2-hydroxy-3-methoxy-1,4-benzoquinone	-	-	-	-	-	0.2 ± 0.1
L	2,3-dimethoxy-5-methyl-1,4-benzoquinone	3.9 ± 2	-	1.1	-	-	1.3 ± 0.2
M	2,3-dimethoxy-5-methylhydroquinone + methyl-paraben	0.3 ± 0.1	-	-	-	-	-
N	methyl-paraben	-	-	-	-	-	0.8 ± 0.2
O	dimethoxy-hydroxy-benzoquinone isomer	-	-	-	-	-	0.2 ± 0.1
P	3-hydroxy-5-methoxy-5-methyl-1,4-benzoquinone	0.3 ± 0.3	-	-	-	-	5.4 ± 0.6
Q	2-phenylphenol	1.1 ± 4.1	-	-	-	-	-
R	unidentified	-	-	-	-	-	0.6 ± 0.2
S	unidentified	-	-	-	-	-	1.2 ± 0.1
T	dimethoxy-hydroxy-methyl-benzoquinone isomer 1	0.1 ± 0.2	-	-	-	-	13.6 ± 1.6
U	dimethoxy-hydroxy-methyl-benzoquinone isomer 2	trace	-	-	-	-	1.8 ± 0.2
V	dimethoxy-hydroxy-methyl-benzoquinone isomer 3	trace	-	-	-	-	0.7 ± 0.1
W	C_14_H_13_O_3_	trace	-	0.1	2.0 ± 1.7	1.1 ± 0.7	-

*Compounds are given as % peak area of whole secretion as described in material & methods; thus, each column represents the chemical secretion profile of a species. Specific profiles (including means and standard variations for each compound) are based on the examination of 23 individuals for *Brachyiulus lusitanus*, a pooled extract (3 individuals) of *Megaphyllum fagorum*, 1 individual of *Cylindroiulus* sp., 24 individuals of *Styrioiulus pelidnus*, 23 individuals of *Styrioiulus styricus*, and 10 individuals of *Typhloiulus* n. sp. Phenolic compounds in bold

## Discussion

### Phenols in Juliformian Millipedes?

Although *p*-cresol and phenol have been reported sporadically as minor compounds in the quinone-rich secretions of julids, such as *Unciger transsilvanicus* (Uncigerini) (Sekulić et al. 2014), and are indicated for several species of *Serboiulus* (Leptoiulini), we here provide the first examples for clearly phenol-based chemical secretions (= phenolic content of secretion >20 %) in the family Julidae. In three species analyzed, phenolic content was higher than 90 %: in *S. pelidnus* and *S. styricus* about 95 %, and in *M. fagorum* the secretions were exclusively phenolic. Most interestingly, these phenol-producing species are not closely related, and the distribution of phenols does not follow an obvious taxonomic pattern. Species with phenolic secretions appear to be unexpectedly present in genera or tribes whose “normal” representatives rely on the common benzoquinonic chemistry. We found such “aberrant”, phenol-producing species in 5 genera, belonging to 3 different tribes of julids - Brachyiulini, Cylindroiulini, and Leptoiulini. So far, each of these tribes was known for uniform benzoquinonic chemistry. Taking the example of Cylindroiulini, hitherto analyzed species of *Cylindroiulus* (Huth 2000), *Enantiulus* (Huth 2000), and *Allajulus* (Bodner and Raspotnig 2012) showed non-phenolic, benzoquinone-dominated secretions. Comparably, preliminary data indicate that the mono-specific cylindroiuline genus *Kryphioiulus* exclusively disharges benzoquinones as well. Thus, phenols appear to have arisen at least twice in cylindroiulines, once in *Styrioiulus* and a second time in a lineage of *Cylindroiulus*. In this respect, it is important to recall the paper of Kluge and Eisner (1971), who reported on a phenol-rich secretion in the parajulid *Oriulus venustus.* Until now, this was the only report on a phenol-predominated secretion in the Juliformia. Assessing this report in light of new data, there is evidence that phenolic secretions are not exceptional in the Julida. Taking current data on julidan secretions as a basis (including literature data), we can currently list 7 species with phenol-dominated secretions out of 57 chemically investigated species. These belong to families Julidae, Blaniulidae, and Parajulidae (Table 1). If we include those species with phenols as minor compounds (3 additional species, Table 1), then phenol-producers comprise about 18 % of the Julida so far investigated. These data indicate that phenolic secretions among the Julida are no rarity and that they are to be expected in a number of additional taxa.

### Phenol Biosynthesis and Evidence for a Shared Phenol Origin

Both benzoquinones and phenols are widespread compounds in the defensive secretions of arthropods. High selective pressure may have led to independent evolution of such compounds in groups such as Coleoptera, Blattaria, Dermaptera, Opiliones, and Diplopoda (Blum 1981, 1996). Additionally, within distinct taxa, it frequently is not clear whether phenolics and benzoquinones may have arisen independently in different sub-taxa, or whether they share a common ancestry (e.g., Caetano and Machado 2013; Raspotnig et al. 2015). In order to address such questions, information on the biosynthetic pathways leading to the compounds is important (Blum 1981). There are only a few studies dealing with the biosynthesis of phenols and benzoquinones in arthropods. In harvestmen, for instance, the biosynthesis of phenols and benzoquinones follows a common multi-step pathway, in the course of which the condensation of acetate and propionate units leads to phenols that subsequently give rise to benzoquinones via para-oxidation (Raspotnig et al. 2015; Rocha et al. 2013). Insects may produce benzoquinones differentially, but nevertheless retain the step of phenol oxidation: for example, the tenebrionid beetle *Eleodes longicollis* produces 1,4-benzoquinone by oxidation of dihydroxybenzene (1,4-hydroquinone), which in turn arises from arbutin, a hydroquinone-glucopyranosid (Happ 1968). Regarding millipedes, it has been assumed that benzoquinone synthesis relies on the availability of aromatic amino acids (Blum 1981). The polydesmidan *Oxidus gracilis*, for instance, produces HCN, benzaldehyde, but also phenol, guaiacol, and arbutin from tyrosine by tyrosine phenol lyase (Duffey and Blum 1977). Based on the occurrence of phenol tyrosine lyase in juliformian millipedes, Duffey and Blum (1977) suggested a similar biosynthetic pathway to juliformian benzoquinones, basically leading to phenolics, then to hydroquinones via arbutin, then to benzoquinones via paraoxidation.

### Evidence for Ancestral Chemical Equipment?

Even though the putative sister group of the Juliformia is still in discussion (Enghoff 1984; Sierwald et al. 2003) there is evidence that close juliformian outgroups may be represented by Nematophora (comprising Callipodida, Stemmiulida, and Chordeumatida) and Polydesmida (Blanke and Wesener 2014; Miyazawa et al. 2014; Sierwald and Bond 2007). Interestingly, in these outgroups, the production of *p*-cresol is widespread or even characteristic (Shear 2015). Phenol biosynthesis requires a complex machinery of different enzymes, making the multiple independent development of the same compounds in closely related taxa less likely. Following this argument, phenolics may be considered the common, ancestral chemical components of juliformian, nematophoran, and polydesmidan defensive secretions. Particularly callipodidan secretions (generally phenol as minor, and *p*-cesol as major constituent) are reminiscent of findings for the Julidae (Ćurčić et al. 2009; Eisner et al. 1963; Makarov et al. 2011; Shear 2015; Shear et al. 2007, 2010).

With respect to Juliformia and to Julida in particular, we thus hypothesize that benzoquinones arose from the ancestral state of phenolics, and that the oxidation-step to benzoquinones evolved later, possibly in early juliformians. This event led to the replacement of phenolics by quinones in most juliformian taxa. Benzoquinones thus may be younger than phenolics, and possibly arose in early juliformians before the split into orders Spirostreptida, Spirobolida, and Julida. This situation basically supports a scenario for diplopod chemosystematics as recently discussed by Shear (2015).

We thus consider the most likely explanation for the patchy, non-taxonomic distribution of phenol-based secretions across the Julida (and presumably in the remaining Juliformia) to be the result of the loss of the para-oxidation step from phenols to benzoquinones, i.e., the last step in the proposed common multi-step pathway to benzoquinone formation (Raspotnig et al. 2015). This loss may occur with the inactivation of a single enzyme, and is thus a parsimonious explanation compared to the possibility of multiple independent evolutions of multi-step pathways to phenolics in different juliformian taxa.

## Electronic supplementary material

Supplemental Table S1 (DOC 115 kb)

## References

[CR1] Blanke A, Wesener T (2014). Revival of forgotten characters and modern imaging techniques help to produce a robust phylogeny of the diplopoda (arthropoda, myriapoda). Arthropod Struct Dev.

[CR2] Blum MS (1981). Chemical defenses of arthropods.

[CR3] Blum MS (1996). Semiochemical parsimony in the arthropoda. Annu Rev Entomol.

[CR4] Bodner M, Raspotnig R (2012). Millipedes that smell like bugs: (E)-alkenals in the defensive secretion of the julid diplopod *Allajulus dicentrus*. J Chem Ecol.

[CR5] Bray JR, Curtis JT (1957). An ordination of the upland forest communities of Southern Wisconsin. Ecol Monographs.

[CR6] Budzikiewicz H, Djerassi C, Williams DH (1967). Mass spectrometry of organic compounds.

[CR7] Caetano D, Machado G (2013). The ecological tale of gonyleptidae (arachnida, opiliones) evolution: phylogeny of a neotropical lineage of armoured harvestmen using ecological, behavioural and chemical characters. Cladistics.

[CR8] Ćurčić BPM, Makarov SE, Tešević VV, Jadranin MB, Vujisić LV (2009). Identification of secretory compounds from the European callipodidan species *Apfelbeckia insculpta*. J Chem Ecol.

[CR9] Duffey SS, Blum MS (1977). Phenol and guaiacol: biosynthesis, detoxication, and function in a polydesmid millipede, *Oxidus gracilis*. Insect Biochem.

[CR10] Duffey SS, Blum MS, Fales HM, Evans SL, Roncadori RW, Tiemann DL, Nakagawa Y (1977). Benzoyl cyanide and mandelonitrile benzoate in the defensive secretions of millipedes. J Chem Ecol.

[CR11] Eisner T, Hurst JJ, Meinwald J (1963). Defense mechanisms of arthropods. XI. the structure, function, and phenolic secretions of the glands of a chordeumoid millipede and a carabid beetle. Psyche.

[CR12] Eisner T, Alsop D, Hicks K, Meinwald J, Bettini S (1978). Defensive secretions of millipedes. Arthropod venoms, handbook of experimental pharmacology.

[CR13] Enghoff H (1984). Phylogeny of millipedes - a cladistic analysis. J Zool Syst Evol Res.

[CR14] Föttinger P, Acosta LE, Leis HJ, Raspotnig G (2010). Benzoquinone-rich exudates from the harvestman *Pachylus paessleri* (opiliones: gonyleptidae: pachylinae). J Arachnol.

[CR15] Happ GM (1968). Quinone and hydrocarbon production in the defensive glands of *Eleodes longicollis* and *Tribolium castaneum* (coleoptera, tenebrionidae). J Insect Physiol.

[CR16] Hara MR, Cavalheiro AJ, Gnaspini P, Santos DYAC (2005). A comparative analysis of the chemical nature of defensive secretions of gonyleptidae (arachnida: opiliones: laniatores). Biochem Syst Ecol.

[CR17] Heethoff M (2012). Regeneration of complex oil-gland secretions and its importance for chemical defense in an oribatid mite. J Chem Ecol.

[CR18] Huth A (2000). Defensive secretions of millipedes: more than just a product of melting point decrease?. Frag Faunistica.

[CR19] Kluge AF, Eisner T (1971). Defense mechanisms of arthropods. XXVIII. A quinone and a phenol in the defensive secretion of a parajulid millipede. Ann Entomol Soc Am.

[CR20] Makarov SE, Ćurčić BPM, Tešević VV, Jadranin MB, Vujisić LV, Ćurčić SB, Mandić BM, Sekulić TL, Mitić BM (2010). Defensive secretions in three species of polydesmids (diplopoda, polydesmida, polydesmidae). J Chem Ecol.

[CR21] Makarov SE, Curčić BP, Vujisić LV, Jadranin MB, Tešević VV, Vučković IM, Sekulić TL, Ćurčić SB, Mitić BM (2011). Defensive secretions in *Callipodella fasciata* (latzel, 1882; diplopoda, callipodida, schizopetalidae). Chem Biodivers.

[CR22] Meinwald YC, Meinwald J, Eisner T (1966). 1,2-dialkyl-4(3H)-quinazolinones in the defensive secretion of a millipede (*Glomeris marginata*). Science.

[CR23] Meinwald J, Smolanoff J, McPhail AT, Miller RW (1975). Nitropolyzonamine: A spirocyclic nitro compound from the defensive glands of a millipede (*Polyzonium rosalbum*). Tetrahedon Lett.

[CR24] Miyazawa H, Ueda C, Yahata K, Su Z-H (2014). Molecular phylogeny of myriapoda provides insights into evolutionary patterns of the mode in post-embryonic development. Sci Rep.

[CR25] Mori N, Kuwahara Y, Yoshida T, Nishida R (1994). Identification of benzaldehyde, phenol and mandelonitrile from *Epanerchodus japonicus* CARL (polydesmida: polydesmidae) as possible defense substances. Appl Entomol Zool.

[CR26] Noguchi S, Mori N, Higa Y, Kuwahara Y (1997). Identification of *Nedyopus patrioticus* (ATTEMS, 1898) (polydesmida: paradoxosomatidae). secretions as possible defense substances. Appl Entomol Zool.

[CR27] Radulović N, Blagojević P, Palić R (2010). Comparative study of the leaf volatiles of *Arctostaphylos uva-ursi* (L.) spreng. and *Vaccinium vitis-idaea* L. (ericaceae). Molecules.

[CR28] Raspotnig G, Bodner M (2014). Beyond benzoquinones: chemical diversity of defensive secretions in the julida (diplopoda). In: tuf I, Tajovský K (eds.) 16th international Congress of myriapodology book of abstracts.

[CR29] Raspotnig G, Bodner M, Schäffer S, Koblmüller S, Schönhofer A, Karaman I (2015). Chemosystematics in the opiliones (arachnida): A comment on the evolutionary history of alkylphenols and benzoquinones in the scent gland secretions of laniatores. Cladistics.

[CR30] Rocha DFO, Wouters FC, Zampieri DS, Brocksom TJ, Machado G, Marsaioli AJ (2013). Harvestmen phenols and benzoquinones: characterisation and biosynthetic pathway. Molecules.

[CR31] Sakata T, Shimano S, Kuwahara Y (2003). Chemical ecology of oribatid mites III. chemical composition of oil gland exudates from two oribatid mites, trhypochthoniellus sp. and trhypochthonius japonicus (acari: trhypochthoniidae). Exp Appl Acarol.

[CR32] Schildknecht H, Maschwitz U, Wenneis WF (1966). Neue stoffe aus dem wehrsekret der diplopodengattung *Glomeris*. Naturwissenschaften.

[CR33] Sekulić TL, Vujisić LV, Ćurčić BPM, Mandić BM, Antić DŽ, Trifunović SS, Gođevac DM, Vajs VE, Tomić VT, Makarov SE (2014). Quinones and non-quinones from the defensive secretion of *Unciger transsilvanicus* (verhoeff, 1899) (diplopoda, julida, julidae), from Serbia. Arch Biol Sci Belgrade.

[CR34] Shear WA (2015). The chemical defenses of millipedes (diplopoda): biochemistry, physiology and ecology. Biochem Syst Ecol.

[CR35] Shear WA, Jones TH, Miras HM (2007). A possible phylogenetic signal in millipede chemical defenses: the polydesmidan millipede *Leonardes musinjucundus* Shelley & Shear secrets p-cresol and lacks a cyanogenic defense (diplopoda, polydesmida, nearctodesmidae). Biochem Syst Ecol.

[CR36] Shear WA, McPherson IS, Jones TH, Loria SF, Zigler KS (2010). Chemical defense of a troglobiont millipede, *Tetracion jonesi* Hoffman (Diplopoda, Callipodida, Abacionidae). Int J Myriapod.

[CR37] Shear WA, Jones TH, Wesener T (2011). Glomerin and homoglomerin from the North American pill millipede *Onomeris sinuata* (Loomis, 1943) (Diplopoda, Pentazonia, Glomeridae). Int J Myriapod.

[CR38] Sierwald P, Bond JE (2007). Current status of the myriapod class diplopoda (millipedes): taxonomic diversity and phylogeny. Annu Rev Entomol.

[CR39] Sierwald P, Shear WA, Shelley RM, Bond JE (2003). Millipede phylogeny revisited in the light of the enigmatic order siphoniulida. J Zool Syst Evol Res.

[CR40] Suzuki TK, Haga WS, Leal S, Kodama S, Kuwahara Y (1989). Secretion of thrips. IV. identification of β-acaridial from three gall-forming thrips (thysanoptera: phlaeothripidae). Appl Entomol Zool.

[CR41] Taira J, Nakamura K, Higa Y (2003). Identification of secretory compounds from the millipede, *Oxidus gracilis* C.L. Koch (polydesmida: paradoxosomatidae) and their variation in different habitats. Appl Entomol Zool.

[CR42] Vagalinski B, Stoev P, Enghoff H (2015). A review of the millipede genus *Typhloiulus* latzel, 1884 (diplopoda: julida: julidae), with a description of three new species from Bulgaria and Greece. Zootaxa.

[CR43] Van den Dool H, Kratz PD (1963). A generalization of the retention index system including linear temperature programmed gas—liquid partition chromatography. J Chromatogr.

[CR44] Vujisić LV, Makarov SE, Ćurčić BPM, Ilić BS, Tešević VV, Gođevac DM, Vučković IM, Ćurčić SB, Mitić BM (2011). Composition of the defensive secretion in three species of european millipedes. J Chem Ecol.

[CR45] Vujisić LV, Antić DŽ, Vučković IM, Sekulić TL, Tomić VT, Mandić BM, Tešević VV, Ćurčić BP, Vajs VE, Makarov SE (2014). Chemical defense in millipedes (myriapoda, diplopoda): Do representatives of the family blaniulidae belong to the 'quinone' clade?. Chem Biodivers.

[CR46] Wood HC (1864). Descriptions of new species of North American iulidae. Proc Acad Nat Sci USA.

[CR47] Wood WF, Hanke FJ, Kubo I, Carroll JA, Crews P (2000). Buzonamine, a new alkaloid from the defensive secretion of the millipede, *Buzonium crassipes*. Biochem Syst Ecol.

[CR48] Wu X, Buden DW, Attygalle AB (2007). Hydroquinones from defensive secretion of a giant Pacific millipede, *Acladocricus setigerus* (diplopoda: spirobolida). Chemoecology.

