# Supplementary material for: “Quinone Millipedes” Reconsidered: Evidence for a Mosaic-Like Taxonomic Distribution of Phenol-Based Secretions across the Julidae
Source: J Chem Ecol. 2016 Mar 14;42:249–58. doi: 10.1007/s10886-016-0680-4 (PMC4839036; doi:10.1007/s10886-016-0680-4)
Supplement: Supplementary file 1 — (DOC 115 kb) [file 10886_2016_680_MOESM1_ESM.doc]

Supplemental Table S1: Collection of species

| family |  | species | locality | date | specimens | voucher no.* |
| --- | --- | --- | --- | --- | --- | --- |
| Blaniulidae |  | *Blaniulus dollfusi* | A Capela Caaveiro (A Coruña, Spain)  43°24'30.62"N, 8°3'24.96"W; 72m, leg. M. Bodner | Aug. 30, 2014 | 1 ♀ | no voucher deposited |
|  |  | *Proteroiulus fuscus* | Reidenau (Carinthia, Austria)  46°46'17.83"N, 14°16'59.69"E; 892m, leg. M. Bodner | Sept. 22, 2013 | 21 ♀ | no voucher deposited |
| Julidae | Brachyiulini | *Brachyiulus lusitanus* | between Strumyani and Mikrevo (Blagoevgrad District, Bulgaria)  41°37'46.30"N, 23°12'11.17"E; 125m , leg. B. Vagalinski | May 11, 2014  Apr. 16, 2015 | 1 ♂, 3 ♀ 14 ♂, 5 ♀ | NHMW 8649 (1♂)  NHMW 8650 (1♀) |
|  |  | *Megaphyllum bosniense* | Plana Mt., SSE of Pasarel Village (Sofia District, Bulgaria)  42°31'19.27"N, 23°30'52.54"E , 900m, leg. B. Vagalinski | Sept. 25, 2011 | 5 ♂, 23 ♀ | NHMW 8651 (1♂)  NHMW 8652 (1♀) |
|  |  | *Megaphyllum fagorum* | Hyrcan Nature Reserve, SW of Zünqüləş (Astara rayon, Azerbaijan)  38°26'53"N 48°45'35"E; 120m,  leg. D. Antić, H. Reip & F. Walther | March 27, 2015 | 1 ♂, 2 ♀ | FBIZO1110 |
|  |  | *Megaphyllum hercules* | Kresna Gorge, N of Kresna (Blagoevgrad District, Bulgaria),  41°47'44.63"N, 23°09'31.04"E; 241m, leg. B. Vagalinski | Apr. 15, 2012 | 6 ♂, 5 ♀ | HNHM |
|  |  | *Megaphyllum silvaticum* | Reidenau (Carinthia, Austria)  46°46'17.20"N, 14°16'56.24"E; 909m, leg. M. Bodner | May 31, 2014  June 15, 2014 | 1 ♂, 2 ♀  10 ♀ | HNHM |
|  | Cylindroiulini | *Allajulus dicentrus* | Maria Rain (Carinthia, Austria)  46°33'2.48"N, 14°17'49.73"E; 516m, leg. M. Bodner | Oct. 15, 2011  Oct. 22, 2011  Nov. 6, 2011 | 7 ♂, 4 ♀  9 ♂, 12 ♀  8 ♂, 13 ♀ | NHMW 8653 (1♂)  NHMW 8654 (1♀) |
|  |  | *Allajulus molybdinus* | Plabutsch (Styria, Austria)  47°5'16.78"N, 15°23'26.78"E; 615m, leg. M. Bodner | June 20, 2011  Aug. 17, 2012  Aug. 21, 2012  June 09, 2013 | 1 ♂, 1 ♀  1 ♂, 6 ♀  4 ♀  3 ♂, 15 ♀ | NHMW 8655 (1♂)  NHMW 8656 (1♀) |
|  |  | *Cylindroiulus apenninorum* | Tirrenia (Tuscany, Italy)  43°38'14.82"N, 10°17'47.79"E; 8m, leg. M. Bodner  Tirrenia (Tuscany, Italy)  43°37'34.24"N, 10°17'27.47"E; 7m, leg. M. Bodner | May 15, 2013  May 16, 2013  May 15, 2013 | 2 ♂, 25 ♀  1 ♂, 4 ♀  3 ♀ | NHMW 8657 (1♂)  NHMW 8658 (1♀) |
|  |  | *Cylindroiulus boleti* | Eisenstadt, Gloriettewarte (Burgenland, Austria)  47°51'19.17"N, 16°30'29.19"E; 326m, leg. M. Bodner | Apr. 20, 2011  Aug. 2, 2011 | 3 ♂, 3 ♀  5 ♂ | NHMW 8659 (1♂) |
|  |  | *Cylindroiulus broti* | Clavel near Saxel, Saxel Municipal forest (Rhône-Alpes region, France)  46°14'29.22"N, 6°22'56.18"E; 1050m, leg. B. Vagalinski | May 14, 2015 | 2 ♂, 4 ♀ | NHMW 8660 (1♂)  NHMW 8661 (1♀) |
|  |  | *Cylindroiulus caeruleocinctus* | Graz-Eggenberg (Styria, Austria)  47°4'51.17"N, 15°24'2.84"E; 399m, leg. M. Bodner | Oct. 19, 2011  Nov. 04, 2011 | 1 ♂, 6 ♀  1 ♂, 2 ♀ | NHMW 8662 (1♂)  NHMW 8663 (1♀) |
|  |  | *Cylindroiulus luridus* | Reidenau (Carinthia, Austria)  46°46'13.68"N, 14°16'39.64"E; 970m, leg. M. Bodner  Vitosha Mt., Zheleznitsa Village (Sofia District, Bulgaria)  42°32'08.09"N, 23°21'44.85"E; 1135m, leg. B. Vagalinski | May 08, 2011  Oct. 07, 2011 | 6 ♂, 3 ♀  6 ♂, 15 ♀ | NHMW 8664 (1♂)  NHMW 8665 (1♀)  NHMW 8666 (1♂)  NHMW 8667 (1♀) |
|  |  | *Cylindroiulus meinerti* | Reidenau (Carinthia, Austria)  46°46'13.68"N, 14°16'39.64"E; 970m, leg. M. Bodner | Apr. 04, 2011  May 08, 2011 | 1 ♂, 3 ♀  3 ♂, 1 ♀ | NHMW 8668 (1♂) |
|  |  | *Cylindroiulus* sp. | A Capela Caaveiro (A Coruña, Spain)  43°24’30.62’’N, 8°3’24.96’’W; 72m, leg. M. Bodner | Aug. 30, 2014 | 1 ♀ | ZMUC00046996 |
|  |  | *Enantiulus karawankianus* | Warmbad Villach (Carinthia, Austria)  46°35'17.01"N, 13°49'30.22"E; 524m, leg. M. Bodner | May 01, 2014 | 12 ♂, 1 ♀ | NHMW 8669 (1♂) |
|  |  | *Enantiulus nanus* | Passau (Bavaria, Germany)  48°34'40.40"N, 13°28'0.53"E; 364m, leg. M. Bodner | March 29, 2014 | 25 ♂, 37 ♀ | NHMW 8670 (1♂)  NHMW 8671 (1♀) |
|  |  | *Enantiulus transsilvanicus* | Plabutsch (Styria, Austria)  47°5'16.78"N, 15°23'26.78"E; 615m, leg. M. Bodner | Aug. 17, 2012  June 09, 2013 | 1 ♂, 4 ♀  4 ♂, 4 ♀ | NHMW 8672 (1♂) |
|  |  | *Kryphioiulus occultus* | Passau (Bavaria, Germany)  48°34'40.40"N, 13°28'0.53"E; 364m, leg. M. Bodner | March 29, 2014 | 5 ♂, 14 ♀ | NHMW 8673 (1♂)  NHMW 8674 (1♀) |
|  |  | *Styrioiulus pelidnus* | Mairist (Carinthia, Austria)  46°44'57.35"N, 14°23'57.88"E; 493m , leg. M. Bodner | 2011  May 18, 2012  June 23, 2012  Aug. 12, 2012 | 1 ♂, 1 ♀ 1 ♂,  1 (undet.), 1 ♂ 1 ♂ | NHMW 8675 (1♂) |
|  |  |  | Warmbad Villach (Carinthia, Austria)  46°35'17.01"N, 13°49'30.22"E; 524m, leg. M. Bodner | May 1, 2014  Sept. 27, 2014 | 5 ♂, 9 ♀ 3 ♂, 1 ♀ | NHMW 8676 (1♂)  NHMW 8677 (1♀) |
|  |  | *Styrioiulus styricus* | Plabutsch (Styria, Austria)  47°5'16.78"N, 15°23'26.78"E; 615m, leg. M. Bodner | Aug. 17, 2012  Aug. 21, 2012  June 9, 2013  July 4, 2014 | 3 ♂, 9 ♀ 3 ♀ 5 ♀ 2 ♂, 1 ♀ | NHMW 8678 (1♂)  NHMW 8679 (1♀) |
|  | Leptoiulini | *Lamellotyphlus* *sotirovi* | Buronov Ponor Pit, Mt. Miroc, E Serbia  44°33'31.04"N, 22°15'40.56"E; 290m, leg. D. Antić | June 22, 2015 | 5 ♂, 5 ♀ | FBIZO 1120 |
|  |  | *Leptoiulus* *proximus* | Leechwald (Styria, Austria)  47°5'5.38"N, 15°27'47.41"E; 413m, leg. M. Bodner | Nov., 2011 | 5 ♂ | NHMW 8680 (1♂) |
|  |  | *Leptoiulus* *trilineatus* | Plana Mt., above Pasarel Village (Sofia District, Bulgaria),  42°31'19.27"N, 23°30'52.54"E; 900m, leg. B. Vagalinski | Sept. 25, 2011 | 5 ♂, 2 ♀ | NHMW 8681 (1♂) |
|  |  | *Ophiulus pilosus* | Plabutsch (Styria, Austria)  47°5'16.78"N, 15°23'26.78"E; 615m, leg. M. Bodner | June 20, 2011 | 2 ♂ | NHMW 8682 (1♂) |
|  |  | *Serboiulus deelemani* | Vetrena Dupka Cave, Vlasi Village, near Pirot, S Serbia  43°0'11.20"N, 22°37'55.70"E; 561m, leg. D. Antić | July 2014 | 5 ♂, 5 ♀ | FBIZO 1160 |
|  |  | *Serboiulus kresnik* | Gornja Lenovačka Pećina Cave, Lenovac Village, Mt. Tupižnica, E Serbia  43°46'30.71"N, 22°9'34.15"E; 335m, leg. D. Antić | July 2014 | 5 ♂, 5 ♀ | FBIZO 1170 |
|  |  | *Serboiulus lucifugus* | Prekonoška Pećina Cave, Prekonoga Village, near Svrljig, S Serbia  43°22'49.3"N, 22°6'7.7"E; 699m, leg. D. Antić | July 2014 | 5 ♂, 5 ♀ | FBIZO 1180 |
|  |  | *Typhloiulus bureschi* | Svinskata Cave by Lakatnik railway station (Sofia District, Bulgaria)  43°05'17.03"N, 23°22'20.94"E; 480m , leg. B. Vagalinski & P. Mitov | Apr. 4, 2013 | 4 ♀ | NHMW 8683 (1♀) |
|  |  | *Typhloiulus* *georgievi* | Toplya Cave by Golyama Zhelyazna Village (Lovech District, Bulgaria)  42°56'53.88"N, 24°29'15.00"E; 466m , leg. B. Vagalinsk | Nov. 11, 2014 | 4 ♂, 4 ♀ | NHMW 8684 (1♂) |
|  |  | *Typhloiulus lobifer* | Minjera Cave, near Škripa, Brač, Croatia  43°21'41.31"N, 16°36'22.39"E; 203m , leg T. Radja | Sep. 2014 | 4 ♂, 3 ♀ | FBIZO 1130 |
|  |  | *Typhloiulus nevoi* | Petrlaška Pećina Cave, Petrlaš Village, Dimitrovgrad, E Serbia  43°4'28.22"N, 22°47'46.85"E; 697m, leg. D. Antić | June 2014 | 3 ♂, 3 ♀ | FBIZO 1140 |
|  |  | *Typhloiulus orpheus* | Trigrad Gorge, near Dyavolskoto garlo Cave (Smolyan District, Bulgaria)  41°36'54.51"N, 24°22'48.94"E; 1250–1300m, leg. B. Vagalinski | May 27, 2014 | 1 ♂, 2 ♀ | NMNHS (Vagalinski et al., 2015) |
|  |  | *Typhloiulus serborum* | Samar Cave System, Kopajkošara Village, near Niš, SE Serbia  43°26'45.40"N, 21°58'34.50"E; 500m, leg. D. Antić | July 2014 | 2 ♂, 3 ♀ | FBIZO 1150 |
|  |  | *Typhloiulus sp. n.* | between Belitsa and Borovo (Plovdiv District, Laki Municipality, Bulgaria) 41°50'20.94"N, 24°51'35.74"E; 695m, leg. B. Vagalinski & P. Mitov | May 1, 2015 | 2 ♂, 8 ♀ | description in progress (Vagalinski, personal communication) |
|  |  | *Xestoiulus* *imbecillus* | Mairist (Carinthia, Austria)  46°44'57.35"N, 14°23'57.88"E; 493m , leg. M. Bodner | Oct. 2011 | 1 ♂, 7 ♀ | NHMW 8685 (1♂)  NHMW 8686 (1♀) |
|  | Uncigerini | *Unciger foetidus* | Drugovačka Šuma, Smederevo, E Serbia  44°39'36"N, 20°55'48"E; 123m,  leg. T. Sekulić | May 2013 | 5 ♂, 5 ♀ | FBIZO 1190 |
|  |  | *Unciger transsilvanicus* | Vitosha Mt., Zheleznitsa Village (Sofia District, Bulgaria)  42°32'08.09"N, 23°21'44.85"E; 1135m, leg. B. Vagalinski | Oct. 7, 2011 | 1 ♂, 5 ♀ | NHMW 8687 (1♂)  NHMW 8688 (1♀) |
|  | Pachyiulini | *Dolichoiulus hercules* | Monte de Gibralfaro (Andalusia, Spain)  36°43'21"N, 4°24'39"W; 93m,  leg. S. Enzinger & M. Bodner | Nov. 8, 2012  March 16, 2013  March 29, 2013  Dez., 2013 | 4 ♂, 12 ♀  9 ♂, 11 ♀  4 ♂, 6 ♀  21 ♂, 25 ♀ | NHMW 8689 (1♂)  NHMW 8690 (1♀) |
|  |  | *Pachyiulus cattarensis* | Vitosha Mt., Zheleznitsa Village (Sofia District, Bulgaria)  42°32'03.34"N, 23°22'35.46"E;  1053m, leg. B. Vagalinski | Apr. 19, 2012 | 3 ♂, 4 ♀ | no voucher deposited |
|  |  | *Pachyiulus hungaricus* | Plana Mt., SSE of Pasarel Village (Sofia District, Bulgaria)  42°31'06.01"N, 23°30'27.20"E; 978m, leg. B. Vagalinski | May 5, 2012  Oct. 4, 2014 | 6 undet.  2 ♀, 1 ♂ | NHMW 8691 (1♂) |
|  | Ommatoiulini | *Ommatoiulus bipartitus* | A Capela Caaveiro (A Coruña, Spain)  43°24'30.62"N, 8°3'24.96’’W; 72m, leg. M. Bodner  nr. Oia (Pontevedra, Spain)  41°59'12.9"N, 8°52'58.8"W; 61m, leg. M. Bodner | Aug. 30, 2014  Sept. 1, 2014 | 2 ♂, 1 ♀    2 ♂, 1 ♀ | ZMUC00046995 (1 ♂)  ZMUC00046997 (1 ♂) |
|  |  | *Ommatoiulus sabulosus* | Peggau (Styria, Austria)  47°12'24"N, 15°21'02"E; 620m,  leg. M. Bodner | July 24, 2012 | 1 ♂, 6 ♀ | NHMW 8692 (1♂)  NHMW 8693 (1♀) |

*Voucher deposition & museum/institution acronyms: NHMW (Natural History Museum of Vienna), ZMUC (Natural History Museum of Denmark), HNHM (Hungarian Natural History Museum, Budapest), NMNHS (National Museum of Natural History Sofia), FBIZO (Faculty of Biology, Institute of Zoology, University of Belgrade).
